# Treatment suspension due to the coronavirus pandemic and mental health of infertile patients: a systematic review and meta-analysis of observational studies

**DOI:** 10.1186/s12889-023-17628-x

**Published:** 2024-01-13

**Authors:** Elnaz Iranifard, Atefeh Yas, Elahe Mansouri Ghezelhesari, Ali Taghipour, Malihe Mahmoudinia, Robab Latifnejad Roudsari

**Affiliations:** 1 Student Research Committee, Mashhad University of Medical Sciences, Mashhad, Iran; 2 Social Determinants of Health Research Center, Mashhad University of Medical Sciences, Mashhad, Iran; 3 Department of Epidemiology, School of Health, Mashhad University of Medical Sciences, Mashhad, Iran; 4 Maternal and Neonatal Research Center, Mashhad University of Medical Sciences, Mashhad, Iran; 5 Department of Obstetrics and Gynecology, School of Medicine, Mashhad University of Medical Sciences, Mashhad, Iran; 6 Nursing and Midwifery Care Research Center, Mashhad University of Medical Sciences, Mashhad, Iran; 7 Department of Midwifery, School of Nursing and Midwifery, Mashhad University of Medical Sciences, Mashhad, Iran

**Keywords:** Anxiety, Assisted reproductive technology, Covid-19, Depression, Infertility, Mental health, Stress, Meta-analysis

## Abstract

**Background:**

Access to fertility treatments is considered a reproductive right, but because of the quarantine due to the coronavirus pandemic most infertility treatments were suspended, which might affect the psychological and emotional health of infertile patients. Therefore, this study was conducted to review the mental health of infertile patients facing treatment suspension due to the coronavirus pandemic.

**Methods:**

This study was conducted based on the Meta-analysis Of Observational Studies in Epidemiology (MOOSE) guideline. The Web of Science, PubMed, Embase, Scopus, and Cochrane library databases were searched by two independent researchers, without time limitation until 31 December 2022. All observational studies regarding the mental health of infertile patients facing treatment suspension including anxiety, depression, and stress were included in the study. Qualitative studies, editorials, brief communications, commentaries, conference papers, guidelines, and studies with no full text were excluded. Quality assessment was carried out using Newcastle–Ottawa Scale by two researchers, independently. The random effects model was used to estimate the pooled prevalence of mental health problems. Meta-regression and subgroup analysis were used to confirm the sources of heterogeneity.

**Results:**

Out of 681 studies, 21 studies with 5901 infertile patients were systematically reviewed, from which 16 studies were included in the meta-analysis. The results of all pooled studies showed that the prevalence of anxiety, depression, and stress in female patients was 48.4% (95% CI 34.8–62.3), 42% (95% CI 26.7–59.4), and 55% (95% CI 45.4–65), respectively. Additionally, 64.4% (95% CI 50.7–76.1) of patients wished to resume their treatments despite the coronavirus pandemic.

**Conclusion:**

Treatment suspension due to the coronavirus pandemic negatively affected the mental health of infertile patients. It is important to maintain the continuity of fertility care, with special attention paid to mental health of infertile patients, through all the possible measures even during a public health crisis.

**Supplementary Information:**

The online version contains supplementary material available at 10.1186/s12889-023-17628-x.

## Background

Infertility is a worldwide health concern, affecting approximately 168 million people of reproductive age, globally [1]. Infertility is defined as the inability to consume a child after 12 or more months of unprotected intercourse [1]. Involuntary childlessness can be considered a life crisis with a great impact on physical, social, emotional, and psychological aspects of life [1–4]. Social stigma, domestic violence, divorce, decrease in self-esteem, stress, anxiety, and depression are amongst the adverse psychosocial effect of infertility [1, 4–6]. Even though fertility treatments have evolved during the past decades, these procedures often cause patients physical and or mental distress [2, 5, 7]. The emotional tension experienced by infertile women may lead to changes in endocrine system regulation and probably result in adverse pregnancy outcomes [5, 6, 8].

A pandemic occurs when a disease spread worldwide, passing international borders and infecting a large number of people [9]. Pandemics and the measures that are taken to control or suppress them such as patient isolation, social distancing, and quarantine can increase mental distress and perceived risk of disease, which leads to psychological consequences including stress, anxiety, depression, delirium, and even post-traumatic stress disorder [10].

In December 2019, cases of infection with the new coronavirus were reported in Wuhan, China [11]. Soon after, the virus was spread across the world, and in May 2020 it was declared a pandemic by World Health Organization [12, 13]. The majority of people infected with this virus through droplet transmission have mild to moderate symptoms, but in some cases, the severity of symptoms may lead to death [13]. Until now 767,984,989 people were infected by the virus and more than 6.9 million people lost their lives [14]. In addition to physical effects, coronavirus can affect the psychological well-being of individuals [11, 15]. People reported fear of infection and/or death, depression, anger, violence, anxiety, and insecurity as the result of the coronavirus pandemic [11, 16, 17].

In order to disconnect the transmission chain and decrease the pressure on the health system, governments adopted strategies including social distancing, quarantine practices, and postponing non-urgent medical treatments [18–20]. Even though access to fertility treatments is considered a reproductive right [1], due to the coronavirus pandemic and its unknown effect on fertility and pregnancy most fertility treatments were postponed [18, 19]. The European Society of Human Reproduction and Embryology (ESRHE) and the American Society for Reproductive Medicine (ASRM), also recommended the suspension of new ART cycles [21, 22]. For infertile couples, especially those with poor prognoses, "time" is a crucial element, and the treatment suspension can harm their mental health [19, 23–25].

The results of systematic reviews indicate that treatment suspension or postponement has a negative effect on patients' mental health. In a systematic review on the mental health and treatment impacts of covid-19 on neurocognitive disorders, an increase in mental health disorders in patients whose treatments were suspended due to the coronavirus pandemic was reported [26]. Similarly, another systematic review reported a negative relationship between mental health and treatment suspension in cancer patients [27].

As it was mentioned, both infertility and the coronavirus pandemic have negative mental outcomes, so that if the impact of treatment suspension is added, the severity of adverse mental health effects on infertile patients would be increased. Although different studies have been conducted regarding the relationship between treatment suspension due to the coronavirus pandemic and the mental health of infertile patients; to the best of our knowledge, no systematic review has been conducted in this relation. It is noteworthy that two systematic reviews have been published with respect to fertility treatment during the Covid-19 pandemic. One systematic review examined the challenges of oncofertility and fertility preservation treatment and the importance of telemedicine during the Covid-19 pandemic [28]. Another systematic review was conducted on the psychological impact of the Covid-19 pandemic on fertility care, and its finding suggested that the covid-19 pandemic causes negative psychological impacts on fertility care [29]; but because of the heterogeneity of studies, the researchers were not able to perform a meta-analysis. In their review, patients were also heterogeneous, with some studies conducted on patients receiving treatment, and some on patients whose treatment was halted or postponed.

Based on the studies conducted prior to the Covid-19 pandemic [2, 30, 31], it is clear that infertile patients suffer from psychological disorders resulted from their infertility. Also, as it was mentioned, systematic reviews on patients other than those who undergo fertility care, suggest that suspension or postponement of treatment has a negative effect on patients' mental health [26, 27]. Therefore, it seems that infertile patients who face treatment suspension or postponement can be at higher risk for mental disorders. Consequently, the mental health status of an infertile patient, who is undergoing fertility treatment might be different from those who experienced treatment postponement. This difference can affect their quality of life and satisfaction with treatment. Therefore, it was decided to conduct a systematic review in this regard. On the other hand, since meta-analyses help with improvement in precision by summarizing and synthesizing of quantitative data from independent yet comparable studies included in a systematic review [32–35], it will be easier and more practical for the audiences to grasp the results of different studies by viewing the results of meta-analysis. In order to reach a precise, clear and summarized result from the findings of the reviewed studies, this systematic review and meta-analysis was conducted to assess the mental health of infertile patients facing treatment suspension due to the Covid-19 pandemic.

## Materials and methods

To do this study, MOOSE Guidelines for Meta-Analyses and Systematic Reviews of Observational Studies was followed [35]. The protocol is registered in PROSPERO (International prospective register of systematic reviews) under the code of CRD42023399725. Also, the study was approved by the Local Research Ethics Committee, Mashhad University of Medical Sciences, Mashhad, Iran (Code of ethics: IR.MUMS.NURSE.REC.1401.056).

### Search strategy and data sources

Two researchers (EI, AY), independently, searched PubMed, Web of Science, Scopus, PsycINFO, Embase, and Cochrane library databases using keywords including coronavirus, covid-19, sars-cov-2, infertility, assisted reproductive technique, psychological distress, stress, anxiety, depression, psychological status, psychological problems/issues, mental health, suspension, and postponement with no time limit until 31 December 2022 (see Additional File 1). Search results of each database was imported to a library created by Endnote reference management software version 9. The software was also used to manage the studies, including identification and removal of duplicated studies, and screening of the titles and abstracts. References of articles which met the inclusion criteria were also searched manually. Since all the relevant articles found by manual search were already included in the study, no records were added by manual search.

Using appropriate keywords, the search of different databases was conducted. At first, duplicate articles were removed. In the next step, the titles and abstracts of the remaining articles were carefully reviewed and the irrelevant articles were excluded. Then the full text of the remaining articles was sought, and articles without access to the full text were excluded. It must be noted that before the exclusion of articles with no access to the full text (n = 1), the corresponding author was reached and she provided us with the full text. Finally, the full text of the remaining articles was reviewed, and those articles that met our inclusion criteria were reviewed in the data extraction process. Two researchers (EI, AY), independently, assessed inclusion and exclusion criteria for each study.

### Inclusion criteria

- Observational studies including cross-sectional, case–control, or cohort studies regarding the mental health of infertile patients facing treatment suspension,
- Studies published in the English language
- PECO was as follows:
  - Participants: Infertile patients seeking treatment
  - Exposure: Treatment suspension due to the Covid-19 pandemic
  - Comparator: None
  - Outcomes: Mental health of infertile patients including anxiety, depression, and stress.

### Exclusion criteria

- No access to the full text of the articles
- Secondary research including systematic reviews, narrative reviews, scoping and rapid reviews as well as other types of articles including qualitative research reports, commentaries and letters to the editor
- Theses or conference abstracts as well as guidelines
- Observational studies which did not follow PECO criteria such as studies on infertile couples with ongoing treatment or infertile couples experiencing pregnancy during the Covid-19 pandemic, or studies which assessed outcomes other than those specified in PECO.
- Languages other than English

### Quality assessment

The Newcastle–Ottawa Scale (NOS) was used for the quality assessment of the studies. The scale is consisted of three sections including selection, comparability, and outcome (exposure in case–control studies). The maximum score for the scale is nine stars, and for each sections including selection, comparability, and outcome respectively is four, two, and three stars [36, 37] (see Additional File 2). The NOS has no established threshold of quality for the studies based on their scores (stars), but previous studies considered scores of 7 or above as high, 5 and 6 as moderate, and 4 and below as low quality [36, 38–41]. Two researchers (EI, EMG), independently, assessed the quality of studies. They shared their results with each other and in cases of inconsistency; the third and senior researcher (RLR) assessed and scored the study. The result of the quality assessment is shown in Additional File 3. All the assessed studies were of high or moderate quality.

### Data extraction

Full texts of 21 included studies were reviewed and data were extracted by two researchers (EI, MM) working together, and any disagreement was clarified by the third researcher (RLR). Data were extracted based on the pre-prepared checklist including the first author's name, publishing year, country of origin, study design, sample size, mean age of patients, mean infertility duration, data collection tools, outcomes including the prevalence of anxiety, depression, and stress, as well as the total score of quality assessment (See Additional File 4).

### Data analysis

Data analysis was conducted based on the extracted data from the included studies. Extracted data were first tabulated from all 21 studies (See Additional File 4). The pooled prevalence (pooled event rate) of anxiety, depression, and stress was estimated using the random-effects method (DerSimonian and Laird method) [42, 43]. The random-effects meta-analysis approach assumes that the different studies are estimating different, yet related, effects and the effects being estimated in the different studies follow some distribution and allows us to address heterogeneity that cannot be explained by other factors [32]. Considering that variables measured using pooled incidence or prevalence can vary between population characteristics, it has been recommended that meta-analyses are performed using the random-effects model [33, 44].

Only studies that reported either the prevalence or number of affected patients by anxiety, depression, and stress were included in the meta-analysis. Since most studies reported the prevalence of patients who wished to resume treatment, the pooled prevalence was also estimated for this variable. Heterogeneity between studies was assessed by the I-squared statistical index. An I^2^ index greater than 75 indicates high heterogeneity [45]. Meta-regression and subgroup analysis were used to confirm the sources of heterogeneity. For meta-regression, mean age and sample size were used as moderators. It must be noted that variation in tools/instruments used to assess mental health status such as anxiety, depression, and stress in primary studies was a common limitation faced in the meta-analysis. Based on the previous meta-analyses on mental health outcomes [46–51], the research team decided to carry out the meta-analysis, and due to the expected variation in tools used to measure anxiety, depression, and stress; subgroup analysis was also done based on the tools for each mental health issue. It must be noted that based on the availability of data in reviewed studies, it was not possible to perform meta-regression or subgroup analysis on moderators such as causes or severity of infertility or attitudes toward infertility based on the region of studies. For publication bias, an Egger test was performed. Meta-analysis was conducted by two researchers (EI, AT) working together. Comprehensive Meta-Analysis Software Version 2 was used to estimate the pooled prevalence and 95% CI and prediction interval by random effects models. A p-value less than 0.05 was considered significant.

## Results

### Search results

**Fig. 1:**
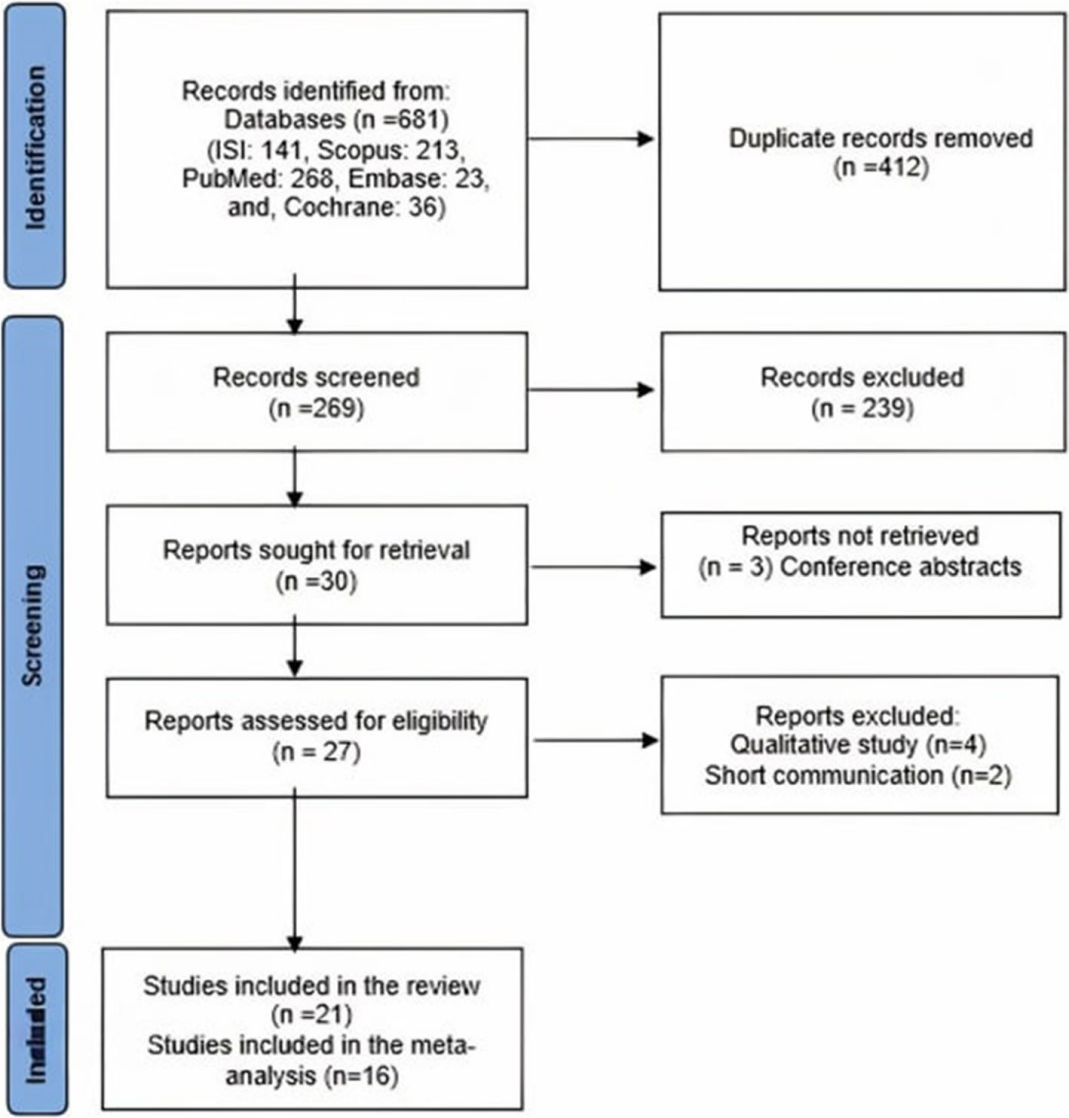
PRISMA Flowchart of study selection

In total 681 studies were identified by searching the databases. After the removal of duplicates, 269 studies were screened for inclusion criteria and 242 studies were excluded. 27 retrieved full-text were assessed for eligibility. It must be noted that one full text was obtained after contacting the corresponding author. Of these, six studies (four qualitative studies and two short communications) did not meet the inclusion criteria, so 21 studies with 5901 participants were included in this systematic review [25, 52–71]. Also, 16 studies were included in the meta-analysis [52–58, 60, 61, 63–66, 68, 70, 71]. The process of study selection is seen in Fig. 1.

### Study characteristics

There was diversity in the region of the studies. Seven studies were from Europe (France [56], Italy [52, 68, 71], Portugal [25], Serbia [59], and Spain [65]); four were from Asia (China [67, 70] and India [54, 55]); four studies were from the Middle East (Iran [60], Israel [63], and Turkey [61, 64]) and six studies were conducted in Canada and/or USA [53, 57, 58, 62, 66, 69]. Except for the study of Dong et al. (2021) and Rasekh Jahromi et al. (2022), which were case–control studies [60, 70], all of the studies had cross-sectional designs. All the participants (n: 5901) were infertile patients seeking treatment during the covid-19 pandemic and their treatment plans were either halted or postponed; the majority of whom were females (90 Percent, n: 5306); and 8.5 percent (n: 504) of the participants were male. Also, 91 participants (1.5 percent) did not mention their gender (Table 1).

**Table 1:** Characteristics of published studies included in the systematic review

*	**1st Author / Year**	**Country**	**Design**	**Sample size**	**Tools**	**Outcome measures**	**Findings**
1	Barra 2020 [52]	Italy	Cross-sectional	524 (308 F, 216 M)	GAD-7 PHQ-9	Anxiety Depression	24% of female patients had anxiety 21% of female patients had depression 37% of patients wished to resume ART treatment
2	Ben-Kimhy 2020 [63]	Israel	Cross-sectional	168 F	Covid-19 anxiety score, MHI-5	Psychological distress	50% of patients had psychological distress 72% of patients wished to resume ART treatment
3	Biviá-Roig 2021 [65]	Spain	Cross-sectional	85 F	HADS	Anxiety Depression	62% of female patients had anxiety 28% of female patients had depression
4	Bortoletto 2021 [66]	USA	Cross-sectional	117 F	HADS	Anxiety Depression	61.5% of female patients had anxiety 28% of female patients had depression 37% of patients wished to resume ART treatment
5	Cao 2021 [67]	China	Cross-sectional	759 F	STAI	Anxiety	Women in the Quarantine zones had a higher tendency to be anxious
6	Cirillo 2021 [68]	Italy	Cross-sectional	140 F	Researcher-made	Anxiety	30% of female patients had anxiety
7	Dillard 2022 [69]	USA	Cross-sectional	304 F	PSS-10	Stress	Patients had a mean score of 19.9 on the Perceived stress scale
8	Dong 2021 [70]	China	Case–control	474 Case (278 F, 196 M)	GAD-7 PHQ-9	Anxiety Depression	34% of female patients had anxiety 43% of female patients had depression
9	Esposito 2020 [71]	Italy	Cross-sectional	627 (588 F, 39 M)	IES-R STAI	Anxiety Stress	72% of female patients had anxiety 65% of patients wished to resume ART treatment
10	Galhardo 2021 [25]	Portugal	Cross-sectional	89 F	DASS-21 PSS-10	Anxiety Depression Stress	Patients had a mean score of 5.1 in the DASS-21 anxiety section Patients had a mean score of 6.7 in the DASS-21 depression section Patients had mean score of 20.9 in Perceived stress scale
11	Gordon 2020 [53]	Canada USA	Cross-sectional	92 F	PHQ-9	Depression	52% of female patients had depression
12	Jaiswal 2022 [54]	India	Cross-sectional	250 F	Self-report PSS-4	Anxiety	72% of female patients had anxiety 98% of patients wished to resume ART treatment
13	Kaur 2020 [55]	India	Cross-sectional	86 (81 F, 5 M)	Researcher-made	Anxiety Depression	11% of female patients had anxiety 14% of female patients had depression 50% of patients wished to resume ART treatment
14	Lablanche 2022 [56]	France	Cross-sectional	421 F	HADS PSS-10	Anxiety Stress	22% of female patients had anxiety 51% of patients had stress 84% of patients wished to resume ART treatment
15	Lawson 2021 [57]	USA	Cross-sectional	787 (648 F, 48 M, 91 N/R)	GAD-7 PHQ-8	Anxiety Depression Stress	71% of female patients had anxiety 77% of female patients had depression 64% of patients had moderate to high distress 41% of patients wished to resume ART treatment
16	Marom-Haham 2021[58]	Canada	Cross-sectional	181 F	MHI-5	Anxiety	60% of female patients had anxiety 82% of patients wished to resume ART treatment
17	Mitrovic 2021 [59]	Serbia	Cross-sectional	176 F	DASS-21	Distress	Perceived threat that COVID-19 poses for infertility treatment had a relationship with general distress
18	Rasekh Jahromi 2022 [60]	Iran	Case control	86 (Case) F	BDI	Depression	60.5% of female patients had depression
19	Sahin 2021 [61]	Turkey	Cross-sectional	220 F	BDI	Depression	65% of female patients had depression
20	Seifer 2021 [62]	USA	Cross-sectional	214 F	STAI-6	Anxiety	Higher stress scores were associated with increased anxiety
21	Tokgoz 2020 [64]	Turkey	Cross-sectional	101 F	STAI, FCV-19S	Anxiety	71% of female patients had anxiety 33% of patients wished to resume ART treatment

Due to the social distancing practice, except for two studies [55, 70], all of the studies were conducted as online surveys [25, 52–54, 56–69, 71]. Also, eight studies used Google forms [52, 58, 60, 61, 63, 65, 68, 69], two used REDCap [62, 66], and two used the SurveyMonkey.com platform [57, 71]. Others did not specify the online measures [25, 53–56, 59, 64, 67, 70]. In terms of data collection tools, except for two studies that used self-structured questionnaires [55, 68], 19 studies used validated instruments [25, 52–54, 56, 58–67, 69–71]. Regarding using specific tools for Covid-19, only two studies used covid-19 related questionnaires, including the Fear of Covid-19 Scale (FCV-19S) and the Covid-19 Anxiety Score [63, 64] (Table 1).

Using the Newcastle–Ottawa scale, seven studies were considered of high quality [52, 57, 60, 65, 67, 69, 71], and 14 studies were of moderate quality [25, 53–56, 58, 59, 61–64, 66, 68, 70] and In regards to quality assessment of cross-sectional studies, all articles (*n* = 19) [25, 52–59, 61–69, 71] achieved maximum score (three stars) in outcome section. While 74% of articles (*n* = 14) [52–54, 56–58, 62, 63, 65–69, 71] achieved maximum score in comparability section and only 10.5% (*n* = 2) [52, 69] received maximum score in selection section. As for case–control studies (*n* = 2) [60, 70], only one study achieved maximum score in Comparability and Exposure section (two and three stars respectively) [60], and both [60, 70] achieved three out of four in selection section. (see Additional File 3).

Based on the findings of this review, the rate of anxiety in infertile women whose treatment was suspended or postponed due to the Covid-19 pandemic ranged from 11 to 72 percent. Also, the prevalence of depression varied from 14 to 77 and the prevalence of stress ranged from 38.9 to 64 percent, which is discussed in more detail. Also, it is important to note that, since the majority of the studies under review did not include male patients in their analysis, meta-analysis could not be performed on male anxiety, depression, and stress due to lack of data.

### Anxiety

Anxiety was the outcome, which was measured in 15 studies [25, 52, 54–58, 62, 64–68, 70, 71]. Different tools including General Anxiety Disorder (GAD-7), State-Trait Anxiety Inventory (STAI, STAI-5, and STAI-6), Hospital Anxiety and Depression Scale (HADS), Mental Health Inventory (MHI-5), and the Depression, Anxiety, and Stress Scale-21 Items (DASS-21) were used in order to measure infertile patients' anxiety. Although Galhardo et al. (2021) found no significant differences regarding anxiety scores between infertile patients with treatment suspension during the coronavirus pandemic and an infertility reference sample [25], Lablanche et al. (2022) reported that the rate of anxiety was much higher than those expected in the infertile population [56]. Two studies reported an increase in anxiety rate in patients who were in confinement [65, 67]. Fear of covid-19 infection and exposure to covid-19 related news were reported to have a negative effect on patients' anxiousness [52, 54]. Being female [52, 71], having previous IVF cycles [52, 67], and older age [52, 54, 64] were also found to increase the anxiety score.

### The pooled prevalence of anxiety in infertile women

**Fig. 2:**
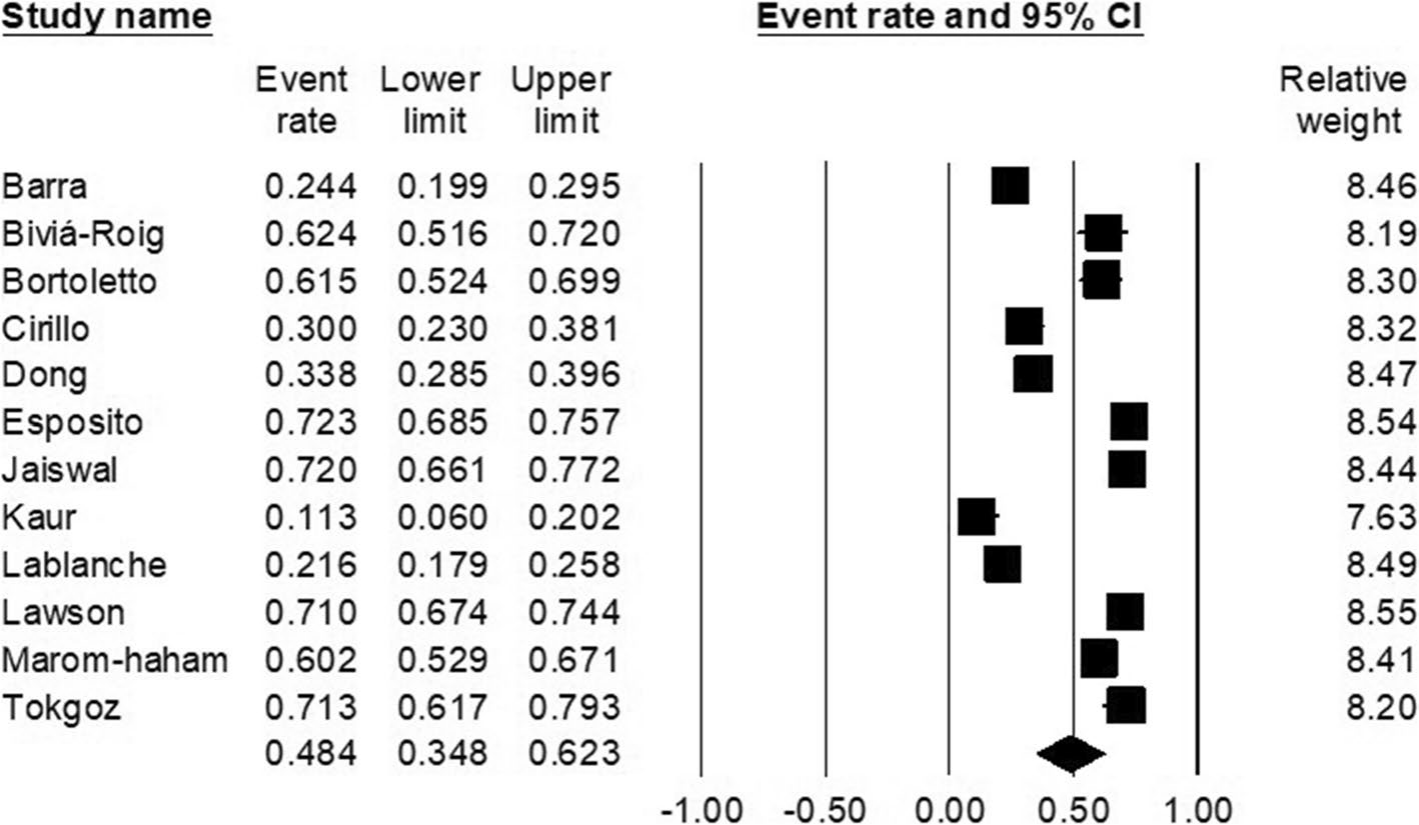
The pooled prevalence of anxiety in female patients

Out of the 15 studies mentioned above, twelve studies reported either the number or percentage of women affected with anxiety during the treatment suspension period. The prevalence of anxiety varied from study to study and it was reported from a low percent of 11 to a high percent of 72. The estimated pooled prevalence was 48.4% (95% CI, 34.8–62.3) (Fig. 2). The I^2^ index was 98.01, which indicated high heterogeneity. Meta-regression was conducted and the sample size was considered as the source of heterogeneity (*p* < 0.001). Publication bias was not observed (Egger test *p*-value: 0.30).

### Subgroup analysis of the prevalence of anxiety

The highest pooled prevalence estimate was calculated across the two studies using the STAI (40, 51), which was 72.1% (95% CI, 68.7–75.4). The lowest estimate was calculated for the three studies using the GAD-7 (32, 39, 45), which was 51.3% (95% CI 48.2–54.4). The heterogeneity was not significant between subgroups (*P* = 0.64) (Table 2).

**Table 2:** Subgroup analysis of the prevalence of anxiety by tools based on random effect analysis

Tool	Number of studies	Prevalence of anxiety	Heterogeneity across studies	
			I^2^	*p* Value
**GAD-7**	3	51.3 (95% CI, 48.2–54.4)	99.03	< 0.001
**HADS**	3	35.9 (95% CI, 31.9–40.2)	97.77	< 0.001
**MHI-5**	1	60.2 (95% CI, 52.9–67.1)	0	1.00
**STAI**	2	72.1 (95% CI, 68.7–75.4)	0	0.83
**Researcher-made**	3	51.8 (95% CI, 46.5–56.9)	97.99	< 0.001
**Heterogeneity between groups**				0.641

### Depression

Depression was measured in 10 studies [25, 52, 53, 55, 57, 60, 61, 65, 66, 70]. Different tools including Patient Health Questionnaire (PHQ-8 and PHQ-9), Beck's Depression Inventory (BDI), Hospital Anxiety and Depression Scale (HADS), Mental Health Inventory (MHI-5), and the Depression, Anxiety, and Stress Scale-21 Items (DASS-21) were used in order to measure infertile patients' depression. Although Galhardo et al. (2021) found no significant differences regarding depression scores between infertile patients with treatment suspension during the coronavirus pandemic and an infertility reference sample [25], Dillard et al. (2022) reported that depressive symptoms were greater during the pandemic [69] and Biviá-Roig et al. (2021) reported an increase in depression score in patients who were in confinement [65]. Also, Rasekh Jahromi et al. (2022) reported that infertile women whose treatment was delayed were more depressed than those who were not under treatment[60]. It was reported that women were more depressed than men [52, 71]. Rasekh Jahromi et al. (2022) and Sahin et al. (2021) both reported a positive correlation between depression and hopelessness [60, 61]; in contrast to Sahin et al. (2021) who found that women with secondary infertility had higher mean depression score [61], Rasekh Jahromi et al. (2022) reported that women with primary infertility were more depressed [60].

### The pooled prevalence of depression in infertile women

**Fig. 3:**
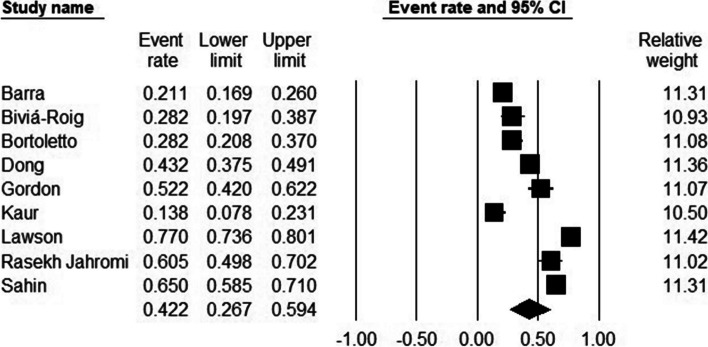
The pooled prevalence of depression in female patients

Out of the 10 studies, nine reported either the number or percentage of women affected with depression during the treatment suspension period. The prevalence of depression varied from study to study and it was reported from a low rate of 14 to a high rate of 77 percent. The estimated pooled prevalence was 42% (95% CI, 26.7–59.4) (Fig. 3). The I^2^ index was 97.70, which indicated high heterogeneity. Meta-regression was conducted and sample size and mean age were considered as the source of heterogeneity (*p* < 0.001). Publication bias was not observed (Egger test *p*-value: 0.09).

### Subgroup analysis of the prevalence of depression

To assess depression, PHQ-9 (32,39,41) with a pooled prevalence of 37.4 (95% CI, 23.8–53.3) was used by three studies. Also, BDI (48, 49) and HADS (34,35) respectively with a pooled prevalence of 62.9 (95% CI, 43.2–79) and 28.2 (95% CI, 14.8–47.2) were used by two studies. Furthermore, PHQ-8 (45) and researcher-made tool (43) each were used in one study. The subgroup analysis suggested evidence of differential prevalence estimates between tools used to assess depression (*P* = 0.001) (Table 3).

**Table 3:** Subgroup analysis of the prevalence of depression by tools based on random effect analysis

Tool	Number of studies	Prevalence of depression	Heterogeneity across studies	
			I^2^	*P* Value
**BDI**	2	62.9 (95% CI, 43.2–79)	0	0.45
**HADS**	2	28.2 (95% CI, 14.8–47.2)	0	0.99
**PHQ-8**	1	77 (95% CI, 53–90.9)	0	1
**PHQ-9**	3	37.4 (95% CI, 23.8–53.3)	95.50	< 0.001
**Researcher-made**	1	13.8 (95% CI, 4.4–35.7)	0	1
**Heterogeneity between groups**				0.001

### Stress

Eleven studies reported stress in infertile patients whose treatments were either suspended or postponed [25, 54, 56–59, 62, 63, 69–71]. Perceived stress scale (PSS-10, PSS-4), Impact of Event Scale-Revised (IES-R), and Depression, Anxiety, and Stress Scale-21 Items (DASS-21) were used to assess stress. Dillard et al. (2022) and Galhardo et al. (2021) reported the mean score of the perceived stress scale-10 in their studies as 19.9 and 20.9 respectively [25, 69]. Three studies reported the prevalence of stress [56, 57, 63]. Higher levels of stress were observed in patients whose treatments were suspended or postponed due to the covid-19 pandemic [69, 70]. Even though two studies reported no significant relationship between demographic characteristics of the patients and stress [58, 69], others reported that age [56, 57, 63], duration of infertility [54, 57], anxiety levels of the patients [56, 58, 62], support system [54, 59], and coping strategies [57, 59] are associated with a higher level of stress.

### The pooled prevalence of stress in infertile women

**Fig. 4:**
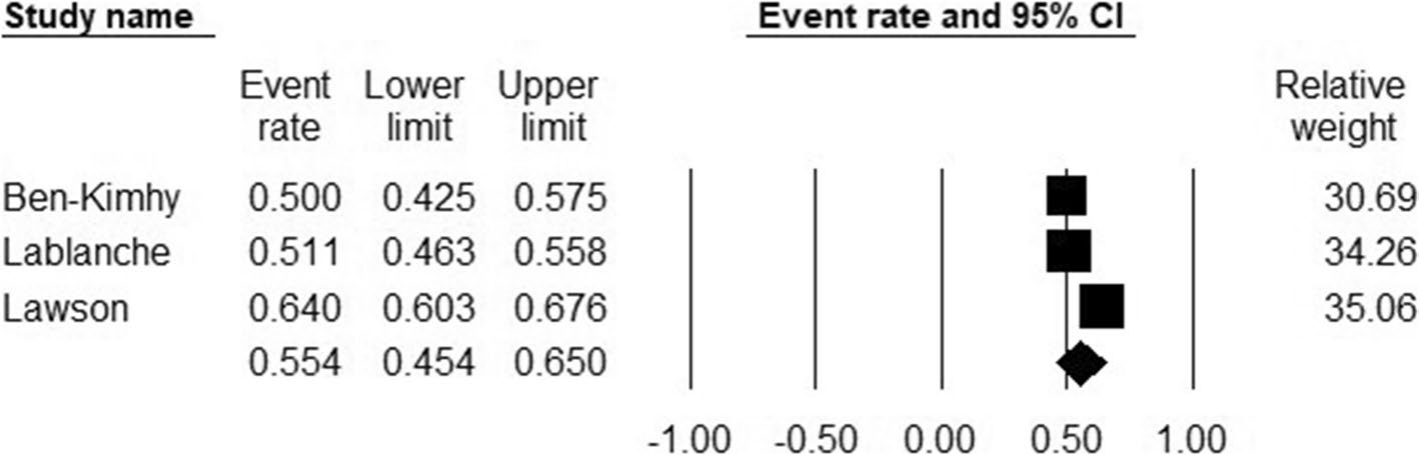
The pooled prevalence of stress in female patients

Out of the 11 studies, three reported either the number or percentage of women affected with stress during the treatment suspension period. The prevalence of stress varied from study to study and it was reported from a low rate of 50 to a high rate of 64 percent. The estimated pooled prevalence was 55% (95% CI, 45.4–65) (Fig. 4). The I^2^ index was 90.99, which indicated high heterogeneity. Publication bias was not observed (Egger test p-value: 0.25). Subgroup analyses and meta-regression were not undertaken because of the small number of studies (n:3) [72].

## Other findings

### The pooled prevalence of patients who wished to resume treatment

**Fig. 5:**
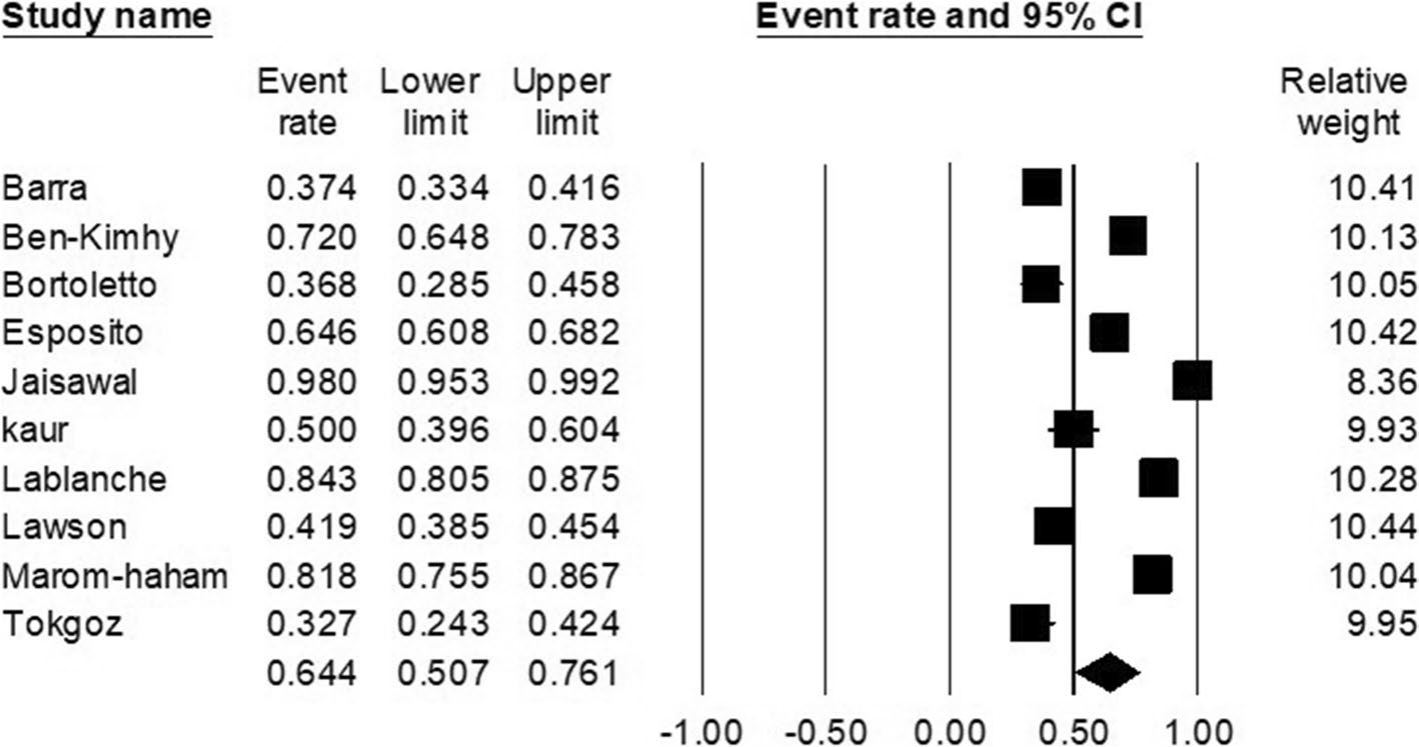
The pooled prevalence of patients who wished to resume treatment

Ten studies reported either the number or percentage of patients who wished to resume infertility treatment [52, 54–58, 63, 64, 66, 71]. The prevalence varied from study to study and it was reported from a low rate of 33 to a high rate of 98 percent. The estimated pooled prevalence was 64.4% (95% CI, 50.7–76.1) (Fig. 5). The I^2^ index was 97.89, which indicated high heterogeneity. Meta-regression was conducted and the sample size was considered as the source of heterogeneity (*p* < 0.001). Publication bias was not observed (Egger test p-value: 0.21).

## Discussion

The results of this review showed that treatment suspension due to the coronavirus pandemic increased the prevalence of anxiety, depression, and stress in female patients. Based on the findings, the rate of anxiety in infertile women whose treatment was suspended or postponed due to the Covid-19 pandemic ranged from 11 to 72 percent. This wide range may be due to variations in tools and cut-off points that were used to measure infertile women's anxiety. A systematic review on the mental health of the general population during the coronavirus pandemic; reported anxiety rates of 6.33%. This finding in comparison to ours, suggests that infertile patients who faced treatment suspension during the covid-19 pandemic had higher rates of anxiety [15]. We estimated a pooled prevalence of 48.4% for anxiety in infertile women facing treatment suspension or postponement, which is higher than the previously reported 36.17% pooled prevalence of anxiety in female infertile patients before the Covid-19 pandemic in 2020 [30]. In accordance with our findings, one study reported that the anxiety rate in infertile women with treatment suspension during the Covid-19 pandemic was much higher than those expected in the infertile population (40). Another study reported a higher level of anxiety in women facing delay in treatment than those who accessed ART treatment during the coronavirus pandemic [70]. The higher prevalence of anxiety can be justified by the increase in anxiety due to both the Covid-19 pandemic and treatment suspension. Although subgroup analysis suggested no significant difference between tools, the HADS scale (anxiety subscale) showed a lower prevalence compared to GAD-7, MHI-5, and STAI. Similarly, another meta-analysis on the prevalence of anxiety in covid-19 patients reported a higher prevalence of anxiety when GAD-7 was used compared to HADS [73]. This can be due to different cut-off points between tools.

In terms of depression, the prevalence varied from 14 to 77 in infertile women whose treatments were either suspended or postponed during the covid-19 pandemic, based on our findings. A systematic review on the mental health of the general population during the coronavirus pandemic reported 14.6 to 48.3 percent of depression [15]. The pooled prevalence of depression before the Covid-19 pandemic was reported at 44.32% in infertile women in low and middle-income countries and 28.03% in high-income countries [31]. In this review, a pooled prevalence of 42% was estimated with majority of the included studies conducted in high-income countries. Comparing our findings to that of the meta-analyses by Kiani et al. (2021) [31], and Xiong et al. (2020) [15]; we observed an increase in depression rate of infertile women whose treatment was suspended or postponed during the Covid-19 pandemic in comparison to both infertile women before the pandemic and the general population during the pandemic. In line with our results, Rasekh Jahromi et al. (2022) also found that during the Coronavirus pandemic the rate of depression were higher in infertile women with treatment suspension than those who were not under treatment [60], however due to the small body of evidence, these findings must be interpreted cautiously. Based on the subgroup analysis, the HADS scale (depression subscale), in comparison to BDI, PHQ-8, and PHQ-9 reported a lower depression prevalence. Similar results were reported in other studies [31, 73]. This difference in prevalence can be explained by the difference in cut-off points of the tools used to measure depression. Also, variations in sample size in different studies must be considered.

A systematic review on the general population during Covid-19 reported the prevalence of stress as 29.6 percent [11]. In our review, the pooled prevalence of stress was 55 percent. Which is higher than those of the general population at the same time. Also, some studies reported a higher level of stress in patients whose treatments were suspended or postponed due to the covid-19 pandemic [69, 70]. Studies reported infertility treatment as a priority for infertile patients and as the top stressor despite an ongoing pandemic [53, 74]. Most of the patients were worried about both the short-term and long-term impact of the treatment suspension on their chances of getting pregnant [59, 64, 66, 68, 69, 75]. In one study a positive relationship was reported between mental distress and the time spent on the coronavirus-related news in infertile patients facing treatment postponement [52]. This positive relationship was also observed in the general population [15].

Based on our findings mental health of patients facing infertility treatment delay, because of the Covid-19 pandemic, was negatively affected. This result is compatible with other studies that stated confinement and treatment suspension have negative effects on the mental health of infertile women [53, 65, 67, 70]. Three studies reported higher levels of mental health distribution in those patients living in the confinement areas [65, 67, 76]; the strict rules and higher exposure to coronaviruses-related news in the confinement areas can be the cause of these findings. As time is considered an important factor in infertility treatment planning, delays, and suspensions were presumed to be a threat to the treatment process [59, 66, 69, 75]. Many infertile patients felt that treatment suspensions were unfair and made them angry [55, 58, 64]. Closure of fertility treatment centers also decreased the quality of life of patients [53, 65, 68]; this is aligned with the findings of a systematic review on the general population [77]. Delay or suspension of treatment due to the coronavirus pandemic was found to be related to increased levels of mental health problems in other patients too. A systematic review reported an increase in mental disorders in patients with neurocognitive disorders whose treatments were suspended [26]. A negative relationship between mental health and treatment suspension in cancer patients was also reported in another systematic review [27]. Maintaining social relationships, receiving support, keeping fit, and having a daily routine could help infertile patients to cope with this situation better [24, 62, 63].

Based on our results 64.4% percent of infertile patients wished to resume their treatment despite the ongoing Covid-19 pandemic. Reports of one study showed that only 6% of infertile patients agreed with delaying their treatment [74]. A cross-sectional study also reported that only 28% of infertile patients were concerned about maternal–fetal transmission of the virus in case of infection during treatment [78]. Based on these findings and in accordance with studies on providing fertility care during covid-19 pandemic [79, 80], it is important to maintain the continuity of fertility care, with special attention paid to mental health of infertile patients, through all the possible measures including virtual care and telemedicine. To substitute the cancelled appointments and ensure patient satisfaction, fertility treatment centers could arrange virtual appointments.

The main limitation of this study was the significant degree of heterogeneity across the studies, which should be taken into account when interpreting the data. The other limitation was that due to the lack of sufficient quantitative data in the reviewed studies, it was not possible to perform a meta-analysis on the relationship between treatment suspension and mental health of infertile patients. Further research with a larger sample size using validated tools is recommended. Also, the short-term and long-term effects of the coronavirus pandemic and treatment suspension on the mental health of infertile patients need to be investigated further.

One of the strengths of this study was that not only it measured the prevalence of anxiety, depression, and stress in infertile women whose treatment were postponed or suspended, but also compared those results in relation to the pre covid-19 pandemic mental health status of infertile women and those of general public during covid-19 pandemic. Also provided quantitative data on the prevalence of patients who wished to resume their treatment. Another strength of this study was the diversity in the included studies in geographical, and socio-economic terms.

## Conclusion

Treatment suspension due to coronavirus pandemic can negatively affect the mental health of infertile patients. Personalized planning could improve infertile patients' mental health. It is important to maintain the continuity of fertility care, with special attention paid to mental health of infertile patients, through all the possible measures including virtual care. Fertility healthcare providers must involve patients in the decision-making process about their treatments even in a public health crisis.

### Supplementary Information

**Additional file 1.** Search Strategy for each database.

** Additional file 2.** The Newcastle-Ottawa Scale.

** Additional file 3.** Quality assessment of the studies based on the Newcastle-Ottawa Scale (NOS).

** Additional file 4.** Data extraction table.

## Data Availability

The datasets used and/or analyzed during the current study are available from the corresponding author on reasonable request.

## Abbreviations

ART: Assisted Reproductive Technology
ASRM: American society of reproductive medicine
BDI: Beck's Depression Inventory
CI: Confidence Interval
DASS-21: Depression, Anxiety, and Stress Scale-21 Items
ESHRE: European Society of Human Reproduction and Embryology
FCV-19S: Fear of Covid-19 Scale
GAD-7: General Anxiety Disorder
HADS: Hospital Anxiety and Depression Scale
IES-R: Impact of Event Scale-Revised
IVF: In Vitro Fertilization
MHI: Mental Health Inventory
MOOSE: Meta-Analysis of Observational Studies in Epidemiology
NOS: Newcastle-Ottawa Scale
PHQ: Patient Health Questionnaire
PSS: Perceived Stress Scale
STAI: State-Trait Anxiety Inventory

## References

[1] world health organization Infertility. https://www.who.int/health-topics/infertility#tab=tab_1. Accessed 24 Sep 2021.

[2] Ying L, Wu LH, Loke AY (2016). The effects of psychosocial interventions on the mental health, pregnancy rates, and marital function of infertile couples undergoing in vitro fertilization: a systematic review. J Assist Reprod Genet.

[3] Bright K, Dube L, Hayden KA, Gordon JL (2020). Effectiveness of psychological interventions on mental health, quality of life and relationship satisfaction for individuals and/or couples undergoing fertility treatment: a systematic review and meta-analysis protocol. BMJ Open.

[4] Abdollahpour S, Taghipour A, Mousavi Vahed SH, Latifnejad Roudsari R (2022). The efficacy of cognitive behavioural therapy on stress, anxiety and depression of infertile couples: a systematic review and meta-analysis. J Obstet Gynaecol.

[5] Frederiksen Y, Farver-Vestergaard I, Skovgård NG, Ingerslev HJ, Zachariae R (2015). Efficacy of psychosocial interventions for psychological and pregnancy outcomes in infertile women and men: a systematic review and meta-analysis. BMJ Open.

[6] Katyal N, Poulsen CM, Knudsen UB, Frederiksen Y (2021). The association between psychosocial interventions and fertility treatment outcome: a systematic review and meta-analysis. Eur J Obstet Gynecol Reproductive Biology.

[7] Ebrahimzadeh Zagami S, Latifnejad Roudsari R, Janghorban R, Mousavi Bazaz SM, Amirian M, Allan HT (2019). Infertile couples’ needs after unsuccessful fertility treatment: a qualitative study. J Caring Sci.

[8] Hassanzadeh Bashtian M, Latifnejad Roudsari R, Sadeghi R (2017). Effects of acupuncture on anxiety in Infertile women: a systematic review of the literature. J Midwifery Reproductive Health.

[9] Phina NF (2011). The classical definition of a pandemic is not elusive. Bull World Health Organ.

[10] Huremovic D. Psychiatry of Pandemics: A Mental Health Response to Infection Outbreak, 1st ed. Psychiatry of Pandemics 2019. 10.1007/978-3-030-15346-5.

[11] Salari N, Hosseinian-Far A, Jalali R, Vaisi-Raygani A, Rasoulpoor S, Mohammadi M, Rasoulpoor S, Khaledi-Paveh B (2020). Prevalence of stress, anxiety, depression among the general population during the COVID-19 pandemic: a systematic review and meta-analysis. Glob Health.

[12] Cucinotta D, Vanelli M (2020). WHO declares COVID-19 a pandemic. Acta Biomed.

[13] World Health Organization Coronavirus. https://www.who.int/health-topics/coronavirus#tab=tab_1. Accessed 16 Aug 2022.

[14] World Health Organization WHO Coronavirus (COVID-19). Dashboard | WHO Coronavirus (COVID-19) Dashboard With Vaccination Data. https://covid19.who.int/. Accessed 16 Jun 2023.

[15] Xiong J, Lipsitz O, Nasri F (2020). Impact of COVID-19 pandemic on mental health in the general population: a systematic review. J Affect Disord.

[16] Sharifi F, Larki M, Latifnejad Roudsari R (2020). COVID-19 outbreak as threat of violence against women. J Midwifery Reproductive Health.

[17] Vindegaard N, Benros ME (2020). COVID-19 pandemic and mental health consequences: systematic review of the current evidence. Brain Behav Immun.

[18] Anifandis G, Messini CI, Daponte A, Messinis IE (2020). COVID-19 and fertility: a virtual reality. Reprod Biomed Online.

[19] Alviggi C, Esteves SC, Orvieto R (2020). COVID-19 and assisted reproductive technology services: repercussions for patients and proposal for individualized clinical management. Reprod Biol Endocrinol.

[20] Larki M, Sharifi F, Manouchehri E, Latifnejad Roudsari R (2021). Responding to the essential sexual and Reproductive Health needs for women during the COVID-19 pandemic: a Literature Review. Malaysian J Med Sci.

[21] American Society for Reproductive Medicine. Patient management and clinical recommendations during the coronavirus (COVID-19) pandemic Patient management and clinical recommendations during the Coronavirus (COVID-19) Pandemic *. 1–9. 2020.

[22] Hambartsoumian E, Nouri K, The ESHRE COVID-19 Working group (2020). A picture of medically assisted reproduction activities during the COVID-19 pandemic in Europe. Hum Reprod Open.

[23] Trinchant RM, Cruz M, Marqueta J, Requena A (2020). Infertility and reproductive rights after the COVID-19 pandemic. Reprod Biomed Online.

[24] Boivin J, Harrison C, Mathur R, Burns G, Pericleous-Smith A, Gameiro S (2020). Patient experiences of fertility clinic closure during the COVID-19 pandemic: appraisals, coping and emotions. Hum Reprod.

[25] Galhardo A, Carolino N, Monteiro B, Cunha M. The emotional impact of the Covid-19 pandemic in women facing infertility. Psychol Health Med. 2021;1–7. 10.1080/13548506.2021.1922721 33913772

[26] Dellazizzo L, Léveillé N, Landry C, Dumais A (2021). Systematic review on the mental health and treatment impacts of COVID-19 on neurocognitive disorders. J Personalized Med.

[27] Dhada S, Stewart D, Cheema E, Hadi MA, Paudyal V (2021). Cancer services during the COVID-19 pandemic: systematic review of Patient’s and Caregiver’s experiences. Cancer Manage Res.

[28] Voultsos PP, Taniskidou A-MI (2021). Fertility treatment during the COVID-19 pandemic: a systematic review. Afr J Reprod Health.

[29] Kirubarajan A, Patel P, Tsang J, Prethipan T, Sreeram P, Sierra S. The psychological impact of the COVID-19 pandemic on fertility care: a qualitative systematic review. Hum Fertility. 2021;1–8. 10.1080/14647273.2021.1938245 34114919

[30] Kiani Z, Simbar M, Hajian S, Zayeri F, Shahidi M, Saei Ghare Naz M, Ghasemi V (2020). The prevalence of anxiety symptoms in infertile women: a systematic review and meta-analysis. Fertility Res Pract.

[31] Kiani Z, Simbar M, Hajian S, Zayeri F (2021). The prevalence of depression symptoms among infertile women: a systematic review and meta-analysis. Fertility Res Pract.

[32] Deeks J, Higgins JP, Altman D. Analysing data and undertaking meta-analyses. In: Higgins JPT, Thomas J, Chandler J, Cumpston M, Li T, Page MJ WV, editor Cochrane Handbook for Systematic Reviews of Interventions version 6.2. Cochrane, 2021;pp 1–649.

[33] Barker TH, Migliavaca CB, Stein C, Colpani V, Falavigna M, Aromataris E, Munn Z (2021). Conducting proportional meta-analysis in different types of systematic reviews: a guide for synthesisers of evidence. BMC Med Res Methodol.

[34] Schwarzer G, Rücker G, Evangelou E, Veroniki AA (2022). Meta-analysis of proportions. Meta-research. Methods in Molecular Biology.

[35] Stroup DF, Berlin JA, Morton SC, Olkin I, Williamson GD, Rennie D, Moher D, Becker BJ, Sipe TA, Thacker SB (2000). Meta-analysis of Observational studies in Epidemiology: a proposal for reporting. JAMA.

[36] Wu XY, Han LH, Zhang JH, Luo S, Hu JW, Sun K (2017). The influence of physical activity, sedentary behavior on health-related quality of life among the general population of children and adolescents: a systematic review. PLoS ONE.

[37] Xu S, Wan Y, Xu M, Ming J, Xing Y, An F, Ji Q (2015). The association between obstructive sleep apnea and metabolic syndrome: a systematic review and meta-analysis. BMC Pulm Med.

[38] Jawad M, Vamos EP, Najim M, Roberts B, Millett C (2019). Impact of armed conflict on cardiovascular disease risk: a systematic review. Heart.

[39] Jenkins RH, Vamos EP, Taylor-Robinson D, Millett C, Laverty AA (2021). Impacts of the 2008 Great recession on dietary intake: a systematic review and meta-analysis. Int J Behav Nutr Phys Activity.

[40] Fernández-de-las-Peñas C, Navarro-Santana M, Gómez-Mayordomo V, Cuadrado ML, García-Azorín D, Arendt-Nielsen L, Plaza-Manzano G (2021). Headache as an acute and post-COVID-19 symptom in COVID-19 survivors: a meta-analysis of the current literature. Eur J Neurol.

[41] Hariyanto TI, Halim DA, Jodhinata C, Yanto TA, Kurniawan A (2021). Colchicine treatment can improve outcomes of coronavirus disease 2019 (COVID-19): a systematic review and meta-analysis. Clin Exp Pharmacol Physiol.

[42] DerSimonian R, Laird N (1986). Meta-analysis in clinical trials. Control Clin Trials.

[43] Borenstein M, Hedges LV, Higgins JPT, Rothstein HR (2010). A basic introduction to fixed-effect and random-effects models for meta-analysis. Res Synthesis Methods.

[44] Saha S, Chant D, Mcgrath J (2008). Meta-analyses of the incidence and prevalence of schizophrenia: conceptual and methodological issues. Int J Methods Psychiatr Res.

[45] Higgins JPT, Thompson SG (2002). Quantifying heterogeneity in a meta-analysis. Stat Med.

[46] Pacheco JPG, Bunevicius A, Oku A (2023). Pooled prevalence of depressive symptoms among medical students: an individual participant data meta-analysis. BMC Psychiatry.

[47] Kiani Z, Fakari FR, Hakimzadeh A, Hajian S, Fakari FR, Nasiri M (2023). Prevalence of depression in infertile men: a systematic review and meta-analysis. BMC Public Health.

[48] Pilania M, Yadav V, Bairwa M, Behera P, Gupta SD, Khurana H, Mohan V, Baniya G, Poongothai S (2019). Prevalence of depression among the elderly (60 years and above) population in India, 1997–2016: a systematic review and meta-analysis. BMC Public Health.

[49] Hawcroft C, Hughes R, Shaheen A, Usta J, Elkadi H, Dalton T, Ginwalla K, Feder G (2019). Prevalence and health outcomes of domestic violence amongst clinical populations in arab countries: a systematic review and meta-analysis. BMC Public Health.

[50] Chau SWH, Wong OWH, Ramakrishnan R (2021). History for some or lesson for all? A systematic review and meta-analysis on the immediate and long-term mental health impact of the 2002–2003 severe Acute Respiratory Syndrome (SARS) outbreak. BMC Public Health.

[51] Ayano G, Shumet S, Tesfaw G, Tsegay L (2020). A systematic review and meta-analysis of the prevalence of bipolar disorder among homeless people. BMC Public Health.

[52] Barra F, La Rosa VL, Vitale SG, Commodari E, Altieri M, Scala C, Ferrero S (2020). Psychological status of infertile patients who had in vitro fertilization treatment interrupted or postponed due to COVID-19 pandemic: a cross-sectional study. J Psychosom Obstet Gynecol.

[53] Gordon JL, Balsom AA (2020). The psychological impact of fertility treatment suspensions during the COVID-19 pandemic. PLoS ONE.

[54] Jaiswal P, Mahey R, Singh S, Vanamail P, Gupta M, Cheluvaraju R, Sharma JB, Bhatla N (2022). Psychological impact of suspension/postponement of fertility treatments on infertile women waiting during COVID pandemic. Obstet Gynecol Sci.

[55] Kaur H, Pranesh G, Rao K (2020). Emotional impact of delay in fertility treatment due to COVID-19 pandemic. J Hum Reproductive Sci.

[56] Lablanche O, Salle B, Perie M-A, Labrune E, Langlois-Jacques C, Fraison E (2022). Psychological effect of COVID-19 pandemic among women undergoing infertility care, a French cohort – PsyCovART psychological effect of COVID-19: PsyCovART. J Gynecol Obstet Hum Reprod.

[57] Lawson AK, McQueen DB, Swanson AC, Confino R, Feinberg EC, Pavone ME (2021). Psychological distress and postponed fertility care during the COVID-19 pandemic. J Assist Reprod Genet.

[58] Marom Haham L, Youngster M, Kuperman Shani A, Yee S, Ben-Kimhy R, Medina-Artom TR, Hourvitz A, Kedem A, Librach C (2021). Suspension of fertility treatment during the COVID-19 pandemic: views, emotional reactions and psychological distress among women undergoing fertility treatment. Reprod Biomed Online.

[59] Mitrović M, Kostić JO, Ristić M. Intolerance of uncertainty and distress in women with delayed IVF treatment due to the COVID-19 pandemic: the mediating role of situation appraisal and coping strategies. J Health Psychol. 201;135910532110499. 10.1177/13591053211049950 34670414

[60] Rasekh Jahromi A, Daroneh E, Jamali S, Ranjbar A, Rahmanian V (2022). Impact of COVID-19 pandemic on depression and hopelessness in infertile women. J Psychosom Obstet Gynecol.

[61] Şahin B, Şahin B, Karlı P, Sel G, Hatırnaz Ş, Kara OF, Tinelli A (2021). Level of depression and hopelessness among women with infertility during the outbreak of COVID-19: a cross-sectional investigation. Clin Exp Obstet Gynecol.

[62] Seifer DB, Petok WD, Agrawal A, Glenn TL, Bayer AH, Witt BR, Burgin BD, Lieman HJ (2021). Psychological experience and coping strategies of patients in the Northeast US delaying care for infertility during the COVID-19 pandemic. Reprod Biol Endocrinol.

[63] Ben-Kimhy R, Youngster M, Medina-Artom TR, Avraham S, Gat I, Marom Haham L, Hourvitz A, Kedem A (2020). Fertility patients under COVID-19: attitudes, perceptions and psychological reactions. Hum Reprod (Oxford England).

[64] Tokgoz VY, Kaya Y, Tekin AB (2020). The level of anxiety in infertile women whose ART cycles are postponed due to the COVID-19 outbreak. J Psychosom Obstet Gynecol.

[65] Biviá-Roig G, Boldó-Roda A, Blasco-Sanz R, Serrano-Raya L, DelaFuente-Díez E, Múzquiz-Barberá P, Lisón JF (2021). Impact of the COVID-19 pandemic on the lifestyles and Quality of Life of Women with fertility problems: a cross-sectional study. Front Public Health.

[66] Bortoletto P, Applegarth L, Josephs L, Witzke J, Romanski PA, Schattman G, Rosenwaks Z, Grill E (2021). Psychosocial response of infertile patients to COVID-19-related delays in care at the epicenter of the global pandemic. Minerva Obstet Gynecol.

[67] Cao L-B, Hao Q, Liu Y, Sun Q, Wu B, Chen L, Yan L (2021). Anxiety level during the second localized COVID-19 pandemic among quarantined infertile women: a cross-sectional survey in China. Front Psychiatry.

[68] Cirillo M, Rizzello F, Badolato L, De Angelis D, Evangelisti P, Coccia ME, Fatini C (2021). The effects of COVID-19 lockdown on lifestyle and emotional state in women undergoing assisted reproductive technology: results of an Italian survey. J Gynecol Obstet Hum Reprod.

[69] Dillard AJ, Weber AE, Chassee A, Thakur M (2022). Perceptions of the COVID-19 pandemic among women with infertility: correlations with Dispositional Optimism. Int J Environ Res Public Health.

[70] Dong M, Wu S, Tao Y, Zhou F, Tan J (2021). The impact of postponed fertility treatment on the sexual health of infertile patients owing to the COVID-19 pandemic. Front Med.

[71] Esposito V, Rania E, Lico D (2020). Influence of COVID-19 pandemic on the psychological status of infertile couples. Eur J Obstet Gynecol Reproductive Biology.

[72] Higgins JP, Green S, editors. Analysing data and undertaking meta-analyse. Cochrane Handbook for Systematic Reviews of Interventions; 2011.

[73] Deng J, Zhou F, Hou W, Silver Z, Wong CY, Chang O, Huang E, Zuo QK (2021). The prevalence of depression, anxiety, and sleep disturbances in COVID-19 patients: a meta-analysis. Ann N Y Acad Sci.

[74] Vaughan DA, Shah JS, Penzias AS, Domar AD, Toth TL (2020). Infertility remains a top stressor despite the COVID-19 pandemic. Reprod Biomed Online.

[75] Gupta M, Jaiswal P, Bansiwal R, Sethi A, Vanamail P, Kachhawa G, Kumari R, Mahey R (2021). Anxieties and apprehensions among women waiting for fertility treatments during the COVID-19 pandemic. Int J Gynecol Obstet.

[76] Qu P, Zhao D, Jia P, Dang S, Shi W, Wang M, Shi J (2021). Changes in Mental Health of women undergoing assisted Reproductive Technology Treatment during the COVID-19 pandemic outbreak in Xi’an, China. Front Public Health.

[77] Melo-Oliveira ME, Sá-Caputo D, Bachur JA, Paineiras-Domingos LL, Sonza A, Lacerda AC, Mendonça V, Seixas A, Taiar R, Bernardo-Filho M (2021). Reported quality of life in countries with cases of COVID19: a systematic review. Expert Rev Respir Med.

[78] Peivandi S, Razavi A, Shafiei S, Zamaniyan M, Orafaie A, Jafarpour H. (2020) Evaluation of attitude among infertile couples about continuing assisted reproductive technologies therapy during novel coronavirus outbreak. medRxiv 2020.09.01.20186320.

[79] Rosielle K, Bergwerff J, Schreurs AMF (2021). The impact of the COVID-19 pandemic on infertility patients and endometriosis patients in the Netherlands. Reprod Biomed Online.

[80] Grens H, de Bruin JP, Huppelschoten A, Kremer JAM (2022). Fertility workup with video consultation during the COVID-19 pandemic: pilot quantitative and qualitative study. JMIR Formative Research.

