# Supplementary material for: Treatment suspension due to the coronavirus pandemic and mental health of infertile patients: a systematic review and meta-analysis of observational studies
Source: BMC Public Health. 2024 Jan 13;24:174. doi: 10.1186/s12889-023-17628-x (PMC10787415; doi:10.1186/s12889-023-17628-x)
Supplement: Supplementary file 1 — Additional file 1. Search Strategy for each database. [file 12889_2023_17628_MOESM1_ESM.pdf]

Additional File 1. Search Strategy for each database

ISI	
All fields: #1 AND #2 AND #3 AND #4	#1(covid-19 OR coronavirus OR sars-cov-2)<br>#2(infertility OR assisted reproductive technique)<br>#3(psychological distress OR stress OR anxiety OR depression OR psychological status OR psychological problems OR mental health)<br>#4(suspension OR postponement)
PubMed	
All fields: #1 AND #2 AND #3AND #4	#1(covid-19 OR coronavirus OR sars-cov-2)<br>#2(infertility OR assisted reproductive technique)<br>#3(psychological distress OR stress OR anxiety OR depression OR psychological status OR psychological problems OR mental health)<br>#4(suspension OR postponement)
Scopus	
All fields: #1 AND #2 AND #3AND #4	#1(covid-19 OR coronavirus OR sars-cov-2)<br>#2(infertility OR assisted reproductive technique)<br>#3(psychological distress OR stress OR anxiety OR depression OR psychological status OR psychological problems OR mental health)<br>#4(suspension OR postponement)
Embase	
Title, Abstract, Keyword: #1 AND #2 AND #3 AND #4	#1(covid-19 OR coronavirus OR sars-cov-2)<br>#2(infertility OR assisted reproductive technique)<br>#3(psychological distress OR stress OR anxiety OR depression OR psychological status OR psychological problems OR mental health)<br>#4(suspension OR postponement)
Cochrane	
Title, Abstract, Keyword: #1 AND #2 AND #3 AND #4	#1(covid-19 OR coronavirus OR sars-cov-2)<br>#2(infertility OR assisted reproductive technique)<br>#3(psychological distress OR stress OR anxiety OR depression OR psychological status OR psychological problems OR mental health)<br>#4(suspension OR postponement)
