# Supplementary material for: Treatment suspension due to the coronavirus pandemic and mental health of infertile patients: a systematic review and meta-analysis of observational studies
Source: BMC Public Health. 2024 Jan 13;24:174. doi: 10.1186/s12889-023-17628-x (PMC10787415; doi:10.1186/s12889-023-17628-x)
Supplement: Supplementary file 2 — Additional file 2. The Newcastle-Ottawa Scale. [file 12889_2023_17628_MOESM2_ESM.pdf]

Additional File 2. The Newcastle-Ottawa Scale

NOS – Cross-sectional studies

Selection:

1. Representativeness of the sample:
    a. Truly representative of the average in the target population. * (all subjects or random sampling)
    b. Somewhat representative of the average in the target group. * (non-random sampling)
    c. Selected group of users/convenience sample.
    d. No description of the derivation of the included subjects.

2. Sample size:
    a. Justified and satisfactory (including sample size calculation). *
    b. Not justified.
    c. No information provided

3. Non-respondents:
    a. Proportion of target sample recruited attains pre-specified target or basic summary of non-respondent characteristics in sampling frame recorded. *
    b. Unsatisfactory recruitment rate, no summary data on non-respondents.
    c. No information provided

4. Ascertainment of the exposure (risk factor):
    a. Validated tools/ instruments *
    b. Non-validated tools/ instruments
    c. No information provided

Comparability: (Maximum 2 stars)

1. Comparability of subjects in different outcome groups on the basis of design or analysis. Confounding factors controlled.
    a. Data/ results adjusted for relevant predictors/risk factors/confounders e.g., age, sex, etc. **
    b. Data/results not adjusted for all relevant confounders/risk factors/information not provided.

Outcome:

1. Assessment of outcome:
    a. Independent blind assessment using standard tool. **
    b. Unblinded assessment using objective standard tool. *
    c. No description

2. Statistical test:
    a. Statistical test used to analyse the data clearly described, appropriate and measures of association presented including confidence intervals and probability level (p value). *
    b. Statistical test not appropriate, not described or incomplete.

NOS – Case-control studies

Selection

1) Is the case definition adequate?
a) yes, with independent validation ∗
b) yes, e.g., record linkage or based on self-reports
c) no description

2) Representativeness of the cases
a) consecutive or obviously representative series of cases ∗
b) potential for selection biases or not stated

3) Selection of Controls
a) community controls ∗
b) hospital controls
c) no description

4) Definition of Controls
a) no history of disease (endpoint) ∗
b) no description of source

Comparability

1) Comparability of cases and controls on the basis of the design or analysis
a) study controls for ________________ (Select the most important factor.) ∗
b) study controls for any additional factor ∗ (These criteria could be modified to indicate specific control for a second important factor.)

Exposure

1) Ascertainment of exposure
a) structured interview where blind to case/control status ∗
b) interview not blinded to case/control status
c) written self-report or medical record only
d) no description

2) Same method of ascertainment for cases and controls
a) yes ∗
b) no

3) Non-Response rate
a) same rate for both groups ∗
b) non respondents described
c) rate different and no designation
