# Supplementary material for: Treatment suspension due to the coronavirus pandemic and mental health of infertile patients: a systematic review and meta-analysis of observational studies
Source: BMC Public Health. 2024 Jan 13;24:174. doi: 10.1186/s12889-023-17628-x (PMC10787415; doi:10.1186/s12889-023-17628-x)
Supplement: Supplementary file 3 — Additional file 3. Quality assessment of the studies based on the Newcastle-Ottawa Scale (NOS). [file 12889_2023_17628_MOESM3_ESM.pdf]

Additional File 3. Quality assessment of the studies based on the Newcastle-Ottawa Scale (NOS)

-	Authors /Cross-sectional studies	Selection (Maximum: ****)				Comparability (Maximum: **)	Outcome (Maximum: ***)			Total score	Quality
		1*	2*	3*	4*	1**	1**	2*			
1	Barra et al 2020	*	*	*	*	**	*	*		8	High
2	Ben-Kimhy et al 2020	*			*	**	*	*		6	Moderate
3	Biviá-Roig et al 2021	*		*	*	**	*	*		8	High
4	Bortoletto et al 2021	*			*	**	*	*		6	Moderate
5	Cao et al 2021	*	*		*	**	*	*		7	High
6	Cirillo et al 2021	*			*	**	*	*		6	Moderate
7	Dillard et al 2022	*	*	*	*	**	*	*		8	High
8	Esposito et al 2020	*	*		*	**	*	*		7	High
9	Galhardo et al 2020	*			*	*	*	*		5	Moderate
10	Gordon et al 2020	*			*	**	*	*		6	Moderate
11	Jaiswal et al 2022	*			*	**	*	*		6	Moderate
12	Kaur et al 2020	*		*	*	*	*	*		6	Moderate
13	Lablanche et al	*			*	**	*	*		6	Moderate
14	Lawson et al 2021	*	*		*	**	*	*		7	High
15	Marom-Haham et al 2021	*			*	**	*	*		6	Moderate
16	Mitrovic et al 2021	*			*	*	*	*		5	Moderate
17	Sahin et al 2021	*			*	*	*	*		5	Moderate
18	Seifer et al 2021	*			*	**	*	*		6	Moderate
19	Tokgoz et al 2020	*		*	*	*	*	*		6	Moderate
-	Authors /Case-control studies	Selection (Maximum: ****)				Comparability (Maximum: **)	Exposure (Maximum: ***)			Total score	
		1*	2*	3*	4*	1**	1 *	2*	3*		
20	Dong et al 2021	*	*		*	*		*		5	Moderate
21	Rasekh Jahromi et al 2022	*	*		*	**	*	*	*	8	High
