# Supplementary material for: Treatment suspension due to the coronavirus pandemic and mental health of infertile patients: a systematic review and meta-analysis of observational studies
Source: BMC Public Health. 2024 Jan 13;24:174. doi: 10.1186/s12889-023-17628-x (PMC10787415; doi:10.1186/s12889-023-17628-x)
Supplement: Supplementary file 4 — Additional file 4. Data extraction table. [file 12889_2023_17628_MOESM4_ESM.pdf]

Additional File 4. Data extraction table

*	1st Author / Year	Country	Design	Sample size/ Sample	Mean age (F)	Mean infertility duration (Year)	Tools	Outcome measures	Findings	Quality assessment score
1	Barra 2020 [52]	Italy	Cross-sectional	524 (308 F, 216 M)	37.3	3.6	GAD-7 PHQ-9	Anxiety Depression	24% of female patients had anxiety.<br>21% of female patients had depression.<br>37% of patients wished to resume ART treatment.	8
2	Ben-Kimhy 2020 [63]	Israel	Cross-sectional	168 F	37.3	2.6	Covid-19 anxiety score, MHI-5	Psychological distress	50% of patients had psychological distress.<br>72% of patients wished to resume ART treatment.	6
3	Biviá-Roig 2021 [65]	Spain	Cross-sectional	85 F	33.5	3	HADS	Anxiety Depression	62% of female patients had anxiety.<br>28% of female patients had depression.	8
4	Bortoletto 2021 [66]	USA	Cross-sectional	117 F	38.3	2	HADS	Anxiety Depression	61.5% of female patients had anxiety.<br>28% of female patients had depression.<br>37% of patients wished to resume ART treatment.	6
5	Cao 2021 [67]	China	Cross-sectional	759 F	N/R	N/R	STAI	Anxiety	Women in the Quarantine zones had a higher tendency to be anxious.	7
6	Cirillo 2021 [68]	Italy	Cross-sectional	140 F	39.4	N/R	Researcher made	Anxiety	30% of female patients had anxiety.	6
7	Dillard 2022 [69]	USA	Cross-sectional	304 F	N/R	N/R	PSS-10	Stress	Patients had a mean score of 19.9 on the Perceived stress scale.	8
8	Dong 2021 [70]	China	Case-control	474 Case (278 F, 196 M)	34	4.5	GAD-7 PHQ-9	Anxiety Depression	34% of female patients had anxiety.<br>43% of female patients had depression.	5
9	Esposito 2020 [71]	Italy	Cross-sectional	627 (588 F, 39 M)	N/R	N/R	IES-R STAI	Anxiety Stress	72% of female patients had anxiety.<br>65% of patients wished to resume ART treatment.	7
10	Galhardo 2021 [25]	Portugal	Cross-sectional	89 F	35.2	N/R	DASS-21 PSS-10	Anxiety Depression Stress	Patients had a mean score of 5.1 in the DASS-21 anxiety section.<br>Patients had a mean score of 6.7 in the DASS-21 depression section.<br>Patients had mean score of 20.9 in Perceived stress scale.	5
11	Gordon 2020 [53]	Canada USA	Cross-sectional	92 F	34.2	3	PHQ-9	Depression	52% of female patients had depression.	6
12	Jaiswal 2022 [54]	India	Cross-sectional	250 F	29.2	5.8	Self-report PSS-4	Anxiety	72% of female patients had anxiety.<br>98% of patients wished to resume ART treatment.	6
13	Kaur 2020 [55]	India	Cross-sectional	86 (81 F, 5 M)	N/R	N/R	Researcher made	Anxiety Depression	11% of female patients had anxiety.<br>14% of female patients had depression.<br>50% of patients wished to resume ART treatment.	6

14	Lablanche 2022 [56]	France	Cross-sectional	421 F	34	4.8	HADS<br>PSS-10	Anxiety<br>Stress	22% of female patients had anxiety.<br>51% of patients had stress.<br>84% of patients wished to resume ART treatment.	6
15	Lawson 2021 [57]	USA	Cross-sectional	787<br>(648 F, 48 M, 91 N/R)	35.7	N/R	GAD-7<br>PHQ-8	Anxiety<br>Depression<br>Stress	71% of female patients had anxiety.<br>77% of female patients had depression.<br>64% of patients had moderate to high distress.<br>41% of patients wished to resume ART treatment.	7
16	Marom-Haham 2021[58]	Canada	Cross-sectional	181 F	37.7	N/R	MHI-5	Anxiety	60% of female patients had anxiety.<br>82% of patients wished to resume ART treatment.	6
17	Mitrovic 2021 [59]	Serbia	Cross-sectional	176 F	36.7	4.9	DASS-21	Distress	Perceived threat that COVID-19 poses for infertility treatment had a relationship with general distress.	5
18	Rasekh Jahromi 2022 [60]	Iran	Case control	86 (Case) F	34.7	4.6	BDI	Depression	60.5% of female patients had depression.	8
19	Sahin 2021 [61]	Turkey	Cross-sectional	220 F	30.6	4.1	BDI	Depression	65% of female patients had depression.	5
20	Seifer 2021 [62]	USA	Cross-sectional	214 F	35.5	N/R	STAI-6	Anxiety	Higher stress scores were associated with increased anxiety.	6
21	Tokgoz 2020 [64]	Turkey	Cross-sectional	101 F	33.3	5.7	STAI,<br>FCV-19S	Anxiety	71% of female patients had anxiety.<br>33% of patients wished to resume ART treatment.	6
